# Validation and Preference-Based Scoring of the York Binaural Hearing-Related Quality of Life Questionnaire for Young People

**DOI:** 10.1097/AUD.0000000000001713

**Published:** 2025-10-30

**Authors:** Adam J. Pedley, Sarah Somerset, Deborah Vickers, Dan Jiang, Padraig Kitterick

**Affiliations:** 1 NIHR Nottingham Biomedical Research Centre, Nottingham, United Kingdom; 2 Hearing Sciences, School of Medicine, University of Nottingham, Nottingham, United Kingdom; 3 Nottingham Clinical Trials Unit, Nottingham, United Kingdom; 4 Department of Clinical Neurosciences, University of Cambridge, Cambridge, United Kingdom; 5 NIHR Guy’s and St Thomas’ Biomedical Research Centre, London, United Kingdom; 6 NIHR Cambridge Biomedical Research Centre, Biomedical Campus, Cambridge, United Kingdom; 7 Hearing Implant Centre, Guy’s and St. Thomas’ NHS Foundation Trust, London, United Kingdom; 8 Centre for Craniofacial and Regenerative Biology, King’s College London, London, United Kingdom; 9 National Acoustic Laboratories, Sydney, Australia; 10 These authors contributed equally to this work.

**Keywords:** Children, Deaf, Health economics, Hearing loss, Patient, PROMS, Validation

## Abstract

**Objectives:**

The York Binaural Hearing-Related Quality of Life questionnaire for Young People (YBHRQL-Y) is a 3-item measure of hearing-related quality of life devised specifically for use with young people (children aged 8 to 16 years old) with hearing loss. This research had three objectives: (1) to assign numerical values where a higher value corresponds to better perceived overall health status (“health utility weights”) to each of 27 unique combinations of difficulties with speech understanding, sound localization, and listening effort (“hearing health states”); (2) to assess its validity and reproducibility when used with young people with hearing loss; (3) to assess the feasibility of a proxy version designed to be completed by the parents/guardians of young people with hearing loss.

**Design:**

Health utility weights were obtained by conducting time trade-off interviews with a cross-sectional sample of 155 young adults, aged 18 to 24 years old, recruited from social media and UK universities. To assess validity and reproducibility, the YBHRQL-Y and other established instruments measuring functional hearing and hearing-related quality of life in children were administered to young people with hearing loss at two time points, 2 wk apart. In total, 71 children aged 8 to 16 yr old with at least a severe hearing loss took part and were recruited from social media, relevant charities, and support groups in the United Kingdom. The feasibility of obtaining information about the binaural hearing-related quality of life of young people with hearing loss indirectly was assessed by administering a proxy version of the YBHRQL-Y to the parents or guardians of the young people who participated in the research. A total of 71 parents or guardians were recruited from social media, relevant charities, and support groups in the United Kingdom.

**Results:**

The health utility weights elicited from young adults varied monotonically with the level of hearing-related impact described on each of the three dimensions of the YBHRQL-Y, such that the greater the degree of hearing-related impact, the poorer the corresponding health state was judged to be by the respondents. Convergent validity analyses suggested that the domains of the YBHRQL-Y measure the intended constructs and the overall measure relates to the respondent’s health-related quality of life. Test-retest analyses suggested it was reliable and showed good agreement between administrations. Pairwise analysis of responses from the young person with hearing loss and those of their parent/guardian suggested that the proxy measure had poor reliability and poor agreement with the measure administered directly to the young person with hearing loss.

**Conclusions:**

The YBHRQL-Y is a valid and reliable measure of hearing-related quality of life when administered directly to a young person aged 8 to 16 with at least a severe hearing loss. An individual’s preference-based score, derived from the preferences of young adults, successfully integrates information about binaural-related hearing across the domains of speech understanding, sound localization, and listening effort. The combination of brief age-appropriate questions, good psychometric performance across time, and a preference-based scoring method makes the YBHRQL-Y a straightforward means to assess hearing-related quality of life in young people with hearing loss.

## INTRODUCTION

The York Binaural Hearing-Related Quality of Life questionnaire (YBHRQL) is a condition-specific measure of quality of life designed specifically for use with adults with hearing loss (Summerfield et al. 2022). The development and construction of the questionnaire was focused on the need to assess the extent to which the ability to combine information from both ears (binaural hearing) can result in the reduction of difficulties on three key dimensions that are considered to have a meaningful impact on the health status of an individual. The key dimensions, chosen on the basis of qualitative and quantitative research on the quality of life impacts of hearing loss, were the ability to understand speech in noise, the ability to localize the source of a sound, and the effort and fatigue required to listen. The resulting 3-item questionnaire allocates the respondent into one of a possible 125 unique health states (Table 1). It has been extensively validated with different populations, and can be used to inform studies of the clinical effectiveness and the cost effectiveness of interventions that seek to improve binaural hearing (Summerfield & Kitterick 2023).

**TABLE 1.** Comparison of the characteristics of the original YBHRQL and the version described here adapted for use in young people (YBHRQL-Y)

	Original YBHRQL	YBHRQL-Y
Domains	Speech understanding Sound localization Listening effort	Speech understanding Sound localization Listening effort
Time trade-off interviews		
N domains	3	3
Method of describing domains	Name of domain only	Age-appropriate description of a relatable situation
N levels of difficulty per domain	3	3
Method of describing difficulty levels	Detailed description of listening experience	None, some, lots
N health states described/valued	27	27
Final questionnaire		
N questions	3 (1 per domain)	3 (1 per domain)
Method of describing domains	Name of domain only	Age-appropriate description of a relatable situation
N response options per question*	5	5
Method of describing difficulty levels*	Detailed description of listening experience	None, some, lots
N possible respondent health states	125	125

* Both the YBHRQL and YBHRQL-Y present the same three levels of difficulty as described in the valuation studies as the first, third, and fifth response options. The YBHRQL includes the intermediate response options “Between 1 and 3” and “Between 3 and 5” as the second and fourth response options, respectively, while the YBHRQL-Y simply includes intermediate response options without any labels.

YBHRQL-Y, York Binaural Hearing-Related Quality of Life questionnaire for Young People.

The YBHRQL is a preference-based measure; that is, the manner in which a measurement is derived from a respondent’s choices on each of the items of the questionnaire is based on preferences expressed by other people who do not necessarily have the condition of interest to not live in a health state that includes the difficulties reported by the respondents. In the case of the YBHRQL, preferences were elicited for 27 unique health states (defined by the combination of 3 levels of difficulty on 3 health domains) that were subsequently used to assign a health value to an expanded set of 125 health states via interpolation (Table 1). These individuals from whom preferences are elicited are typically sampled such that their preferences can be assumed to represent the degree to which a member of the public would consider a specific set of difficulties to impact on their overall quality of life. A questionnaire developed and scored in such a manner has the advantage that the measurements it provides, and specifically how much those measurements may change after an intervention has been provided, can be directly linked to how the general public views what is important for a good quality of life. The YBHRQL was created as a condition-specific preference-based measure to overcome the issue that many non–condition-specific measures (e.g., HUI3; Furlong et al. 2001) are minimally insensitive to sensory disorders (Longworth et al. 2014) and, in particular, they lack the sensitivity to distinguish changes in the hearing-related quality of life gain between bilateral and unilateral cochlear implantation in adults (Summerfield et al. 2022).

Preference-based methods have also been used in the creation of quality of life measures in children (Stevens 2010). The research described here was conducted to validate an adapted version of the YBHRQL that is suitable for use with young people (the YBHRQL-Y) and to develop a preference-based scoring system for that measure based on the preferences of young adults. YBHRQL-Y (Somerset et al. 2023) was designed specifically to be used in a UK clinical trial that assesses hearing interventions for children (aged 8 to 16 years old) with cochlear implants (Both EARS Training Package “BEARS,” (Vickers et al. 2021)). The requirements set in the adaptation of the adult measure for young people were that it was designed specifically for children, designed specifically for children with hearing loss, and a preference-based measure that produces health states with assigned health utility weights. These health utility values can then be used in the health economic evaluation of the BEARS trial.

Somerset et al. (2023) adapted the YBHRQL for children by conducting interviews with participants aged 8 to 16 years old. They used the Qualitative Pre-test Interview (QPI; (Buschle et al. 2021)) method as a framework to adapt the measure for children. The QPI method is grounded in theory that states patient-reported outcome measures should frame questions in a format that participants can engage with and use appropriate language. It achieves this through “active listening and active understanding and the ability of the researcher to switch between these whilst conversing with participants” (Somerset et al. 2023). In doing so, the QPI method produces questions that are both relevant and relatable to the end user. The resulting questionnaire retained the same three-domain structure as the original YBHRQL but differed in that each domain was described in relation to an everyday situation that would be familiar to the target age group, and a simpler method of describing different levels of difficulty on each domain was used (Table 1).

This article describes research conducted to validate a version of the YBHRQL that is suitable for use with young people, the YBHRQL-Y. Health utility weights for each of the 125 unique health states that the YBHRQL-Y produced were derived by eliciting health state preferences from young people aged 18 to 24 years old. The validity and also the reproducibility (i.e., both reliability and agreement) of the YBHRQL-Y was assessed by administering the YBHRQL-Y, and other related measures, to children with hearing loss at two time points, 2 wk apart.

Given that the YBHRQL-Y was developed using the QPI method with children and uses age-appropriate language and contexts, it was anticipated that a proxy version would not be required within the intended age range of 8 to 16 years old. However, the current research explored the validity of administering a proxy version of the YBHRQL-Y to adults, which may be useful if children lack the communication skills to complete the YBHRQL-Y. These could be children at the younger end of our age range or children with additional needs. Several well-established “gold standard” preference-based measures (e.g., HUI3; Furlong et al. 2001: CHU9D; Stevens 2010) supply proxy versions for adults to complete if children are unable to complete themselves, so this option was explored, even if the method used to develop the YBHRQL-Y (i.e., the QPI method) should ensure this is not required.

## MATERIALS AND METHODS

### Participants

#### Time Trade-Off Interviews

The sample size was determined based on the objective of defining a health utility weight for each of the 27 health states defined by the combination of three levels of difficulty on each of the three domains (speech understanding, sound localization, and listening effort) with sufficient precision to detect differences between health states as small as 0.05 (Horsman et al. 2003; Summerfield et al. 2022). A sample size of 148 participants would allow for the mean utility of each health state to be estimated with a confidence interval (CI) width of 0.048.

A total of 155 young adults aged 18 to 24 (M = 21.1, SD = 1.6) who were sufficiently fluent in the English language to undertake the time trade-off (TTO) interview participated. Participants identified as male (N = 46, 29.5%), female (N = 70, 69.5%), or nonbinary (N = 1, 0.5%). Participants did not require a hearing loss to take part, but this was also not an exclusion criterion. A total of eight participants (5.1%) self-identified as having a diagnosed hearing loss. These hearing losses were categorized as mild (N = 3), moderate (N = 4), and severe (N = 1). Participants received a £20 gift voucher upon completion of the study. The study received a favorable ethical opinion from the Faculty of Medicine and Health Sciences, University of Nottingham (UK, FMHS 53-0822).

Although the YBHRQL-Y was adapted with and designed for use in children aged 8 to 16 yr old, the participants from whom health state utility weights were elicited were aged 18 to 24 yr old. Within TTO interviews, participants must imagine that they have only a limited amount of time to live before certain death, and participants must make judgements about how many years of life they would trade to have better hearing. It was considered inappropriate to ask children to undertake such a task. As an alternative, young adults aged 18 to 24 were considered old enough to undertake a TTO interview but also young enough to remember what it was like to be at school, which is the setting used for all three scenarios described in the YBHRQL-Y. Before the TTO interview, participants were told the process would include discussions about their (imagined) death and were given the option to withdraw consent at any time and leave the study.

#### Reliability and Validity Assessments

A minimum sample size for the reliability and validity assessments of the YBHRQL-Y was determined to be 70 based on 2 considerations. The first consideration was the recommended minimum sample size of 50 participants for conducting assessments of test-retest reliability and agreement (De Vet et al. 2011). The second consideration was that at least 68 participants are required to detect at least modest correlations (*r* = 0.3) of a known direction for the purposes of convergent validity analyses, selected based on the strength of observed associations between the original YBHRQL and generic measures of quality of life (Summerfield et al. 2022).

A total of 71 children aged 8 to 16 (M = 11.6 years, SD = 2.8 years) who were all diagnosed with a bilateral hearing loss (severe loss, N = 14, profound loss, N = 57) and who were sufficiently fluent in the English language to complete the YBHRQL-Y and related measures participated. Of those, 35 had bilateral cochlear implants, 5 had a unilateral cochlear implant (of which 1 used a contralateral hearing aid), and 9 had been fitted with bilateral hearing aids. Participants were recruited from social media, relevant charities, and support groups. Participants received a £40 gift voucher upon completion of the final measure. The parents or guardians of the 71 children were also recruited to complete proxy versions of the YBHRQL-Y and related measures, provided they were sufficiently fluent in the English language to complete the assessments. No reimbursement was offered for their participation. The study received a favorable ethical opinion from the Health Research Authority South Yorkshire Research Ethics Committee (approval reference number 22/YH/0018).

### Procedures

#### TTO Interviews

The study was advertised on social media. Participants expressed an interest to take part in the research by completing an online expression of interest form. Once eligibility was confirmed and consent was received, a 1-hr appointment was planned to conduct the interview. The interviews were conducted by 1 of 4 researchers (3 female, 1 male) who were all trained and conducted several practice interviews before interviewing a participant. To ensure consistency between interviewers, a standard script was produced that interviewers read from throughout the TTO interview based on the interview process developed for the US valuation of the EQ-5D-3L (Agency for Healthcare Research and Quality 2012).

Before the TTO interview, participants completed a number of warm-up tasks. First, participants were introduced to the three domains (i.e., speech understanding, sound localization, and listening effort) and the three difficulty levels for each domain set out in the YBHRQL-Y. Next, participants were asked to imagine they were back at school and to score themselves on the YBHRQL-Y. After this, participants were shown hearing health utility states from the YBHRQL-Y and were asked to rank them from best to worst. Participants were then asked to imagine they had 10 yr left to live and were again asked to rank a selection of hearing health utility states from best to worst. Finally, participants were asked to give an indication of their current general health and to state if they had any known hearing loss. These warm-up tasks deliberately introduced the elements needed for the TTO interview in sequential order; participants were first introduced to the scenarios and difficulty levels within each domain of the YBHRQL-Y before being introduced to the concept of ranking health states and then finally imagining themselves in different health states with a limited number of years to live.

In the next part of the interview, preferences for each of 27 possible health states described by the YBHRQL-Y were elicited using the TTO technique. Although the YBHRQL-Y asks respondents to select from 1 of 5 levels of difficulty on each of its 3 domains, resulting in 125 possible response patterns on the instrument, only 3 of the 5 levels of difficulty are associated with a specific text description with the other 2 options forming intermediate choices between those levels. Thus, a total of 27 health states are explicitly described by the instrument and were included in the TTO interviews. Participants first completed the TTO task for one practice health state (chosen at random) before completing the task for all 27 hearing health states. Health states were presented in a random order, with a comfort break after the 9th and 18th hearing health state valuation. Each TTO interview took approximately 1 hr.

**Fig. 1.**
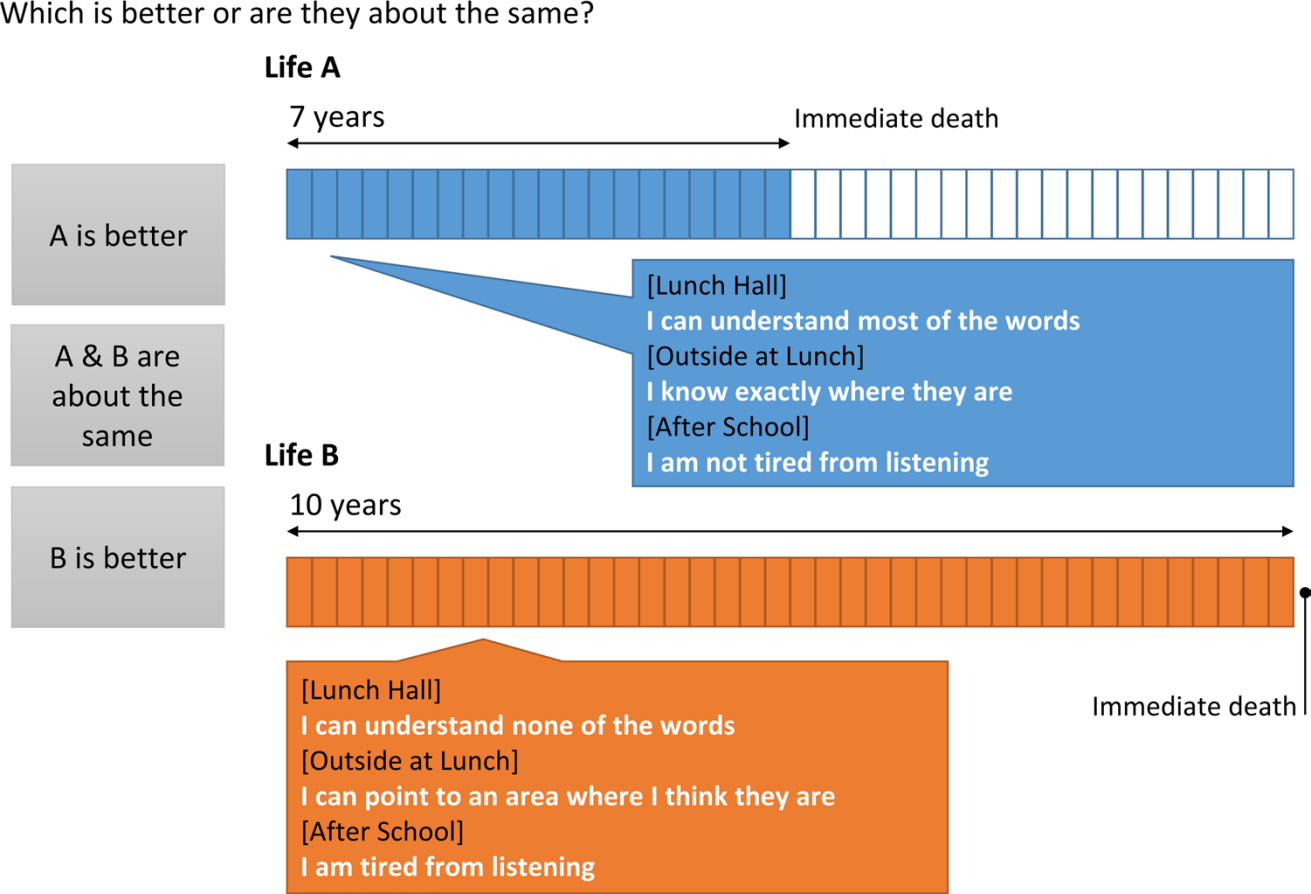
Visual time trade-off board used during the interviews to elicit preferences from young adults for each of 27 health states described in the YBHRQL-Y. YBHRQL-Y indicates York Binaural Hearing-Related Quality of Life questionnaire for Young People.

The conventional TTO methodology was used based on the MVT protocol of the original UK valuation of the EQ-5D-3L (Oppe et al. 2016). Participants were guided through the task using a computerized TTO board that provided a visual representation of the choice they were being asked to make (Fig. 1). The board layout was varied based on whether the state being valued was considered better or worse than death. For states considered better than death, participants were asked to choose between 10 yr in the selected health state and 10 to *x* yr in a state with no difficulty on all three domains followed by immediate death. For states considered worse than death, participants were asked to choose between immediate death and *x* years in the selected health state followed by 10 to *x* yr in the state with no difficulty on all three domains. The value of *x* was initially adjusted from the starting value of 5 yr in 1-yr increments until the first reversal in participants’ preferred option, and then by 6-mo increments until the second reversal. A 3-mo increment or decrement to *x* was then applied based on the response immediately following the second reversal at which point the process terminated. The process was also terminated at any point if the participant considered both choices to be equally preferable.

#### Reliability and Validity Assessments

The parents or guardians of the children expressed an interest for their child to take part in the research by completing an online expression of interest form. Once eligibility was confirmed and consent was received, a link to the parental proxy YBHRQL-Y questionnaire was sent. Once this was completed, a link to the first set of online questionnaires for the child was sent. Thirteen days after completion of these questionnaires, a link to the second set of online questionnaires was sent with the instruction to complete it the next day (i.e., 2 wk after the first administration of each questionnaire). This short period was chosen to minimize the possibility of any major changes in hearing-related quality of life occurring within such a short timeframe. The test and retest questionnaire sets for children included the YBHRQL-Y, the Speech, Spatial and Qualities of Hearing Scale for Children with Impaired Hearing (SSQ-Ch), the Vanderbilt Fatigue Scale for Children with Hearing Loss (VFS-Peds), the Health Utilities Index Mark 3 (HUI3), and the Children’s Health Utility 9-Dimension measure (CHU9D). Parents were instructed not to assist with the children’s responses to ensure that the data could be used to examine the difference between parental and child responses.

The YBHRQL-Y is an adapted version of the YBHRQL, a validated preference-based measure of binaural hearing-related quality of life for use in adults with hearing loss (Summerfield et al. 2022; Summerfield & Kitterick 2023). As with the original version, the YBHRQL-Y asks respondents to indicate the level of difficulty they experience in three domains of hearing: speech perception in noise, localization, and effort and fatigue. Participants choose between five levels of difficulty spanning the range from no difficulty to a description of extreme difficulty appropriate for each domain. For each domain, the adapted version retains the approach of the original version in describing listening situations that are relevant to each domain but uses a simplified response scale suitable for use with participants as young as 8 yr old. The proxy version of the YBHRQL-Y administered to parents and guardians was identical apart from the questions asking about “your child” rather than about “you.”

Analyses of the construct validity of the YBHRQL-Y involved assessing associations between each of the constituent domains and reference measures of the constructs underpinning each domain. The reference measures of difficulty with speech in noise and sound location were derived from the SSQ-Ch (Galvin & Noble 2013). The Speech (10 items) and Spatial Hearing (13 items) domains from the SSQ-Ch were used in determining the construct validity of the related domains of the YBHRQL-Y. The Qualities of Hearing Domain (10 items) was not administered to participants in this study. The reference measure of listening fatigue was the VFS-Peds (Hornsby et al. 2022). This is a 10-item measure that measures fatigue specifically related to children with hearing loss.

The assessment of construct validity also included examining associations between the YBHRQL-Y and two generic measures of quality of life. The HUI3 is a preference-based measure of health-related quality of life (Furlong et al. 2001) that has been widely used in hearing-related studies due to its inclusion of communication in both quiet and noisy listening situations as one of the core domains (Summerfield & Barton 2019). The version used in the current study (self-assessed, self-administered) comprises 15 items measuring 8 dimensions of health (vision, hearing, speech, ambulation, dexterity, emotion, cognition, and pain/complaints) that combine to derive an overall health utility weight based on the preferences of a sample of the Canadian public (Furlong et al. 2001). The CHU9D is another preference-based measure of health-related quality of life but one specifically developed for use in children (Stevens 2010). The nine items measure nine dimensions of health (worry, sadness, pain, tiredness, annoyance, school-work, sleep, self-care, activities) that combine to derive an overall health utility weight based on the preferences of a sample of the adult general population in the United Kingdom.

### Data Analyses

#### TTO Interviews

The number of years that the participant chose to trade (*x*) in states considered better than death was used to derive the health utility for that hearing health state using the formula (10 − *x*)/10, giving a value between 0 (death) and 1 (perfect hearing health) where higher values indicated less disease burden. Utility values for states worse than death were calculated using the formula (*x* − 10)/*x* and scaled so that the lowest possible utility value was −1. The mean health utility assigned by the 155 participants to each of the 27 hearing health states was then calculated. These values were then used to interpolate health utility values for the remaining 98 hearing health states described in the YBHRQL-Y using the intermediate difficulty levels that are not assigned an explicit description using the same approach used for the original YBHRQL (Summerfield et al. 2022). Figure 1 shows a screenshot of the program used to deliver the TTO interview.

#### Reliability and Validity Assessments

Intraclass correlations (ICC) were used to assess the reliability of the continuous health utility values obtained from repeated administrations of the YBHRQL-Y (De Vet et al. 2011). ICC values were classified as indicating poor, moderate, good, or excellent reliability if they were <0.5, 0.5 to 0.75, 0.75 to 0.9, or >0.90 (Koo & Li 2016). Each participant’s responses on the YBHRQL-Y were assigned a health utility value by looking up the corresponding mean utility or extrapolated mean utility derived from the TTO interview study. The reliability of the categorical responses on each of the individual domains (i.e., the specific level of difficulty selected from the five available levels) was assessed using the weighted Cohen’s Kappa coefficient (Vanbelle & Albert 2009). Bland-Altman plots were used to visualize the extent to which the health utility values obtained from repeated administrations of the YBHRQL-Y agreed between test and retest and whether any disagreement was systematic (Bland & Altman 1986). These analyses of reliability and agreement conducted to compare the children’s test and retest responses were also conducted to compare the child and parent responses.

Construct validity was assessed at the level of the individual domains of the YBHRQL-Y and at the level of the overall health utility value. Linear regression models were used to assess the relationship between the categorical responses on the speech in noise domain and the continuous score from the SSQ-Ch speech domain, the categorical responses on the localization domain and the continuous score from the SSQ-Ch spatial hearing domain, and finally, the categorical responses on the effort & fatigue domain and the continuous VFS-Peds score. Regression models were also used to assess the relationship between the continuous YBHRQL-Y health utility values and both the continuous HUI3 health utility values and the continuous CHU9D health utility values.

## RESULTS

### TTO Interviews

No participant assigned a lower value to the most advantageous health state (i.e., no difficulties on all three domains) than the least advantageous health state (i.e., significant difficulties on all three domains), suggesting that judgements were internally consistent. In addition, no participants declined to trade any number of years to return to the most advantageous health state. A total of 21 participants (13.5%) defined at least one hearing health state to be “worse than death.” In all of these hearing health states ranked “worse than death,” at least one of the domains was described in terms of the greatest possible level of difficulty available for that domain.

**Fig. 2.**
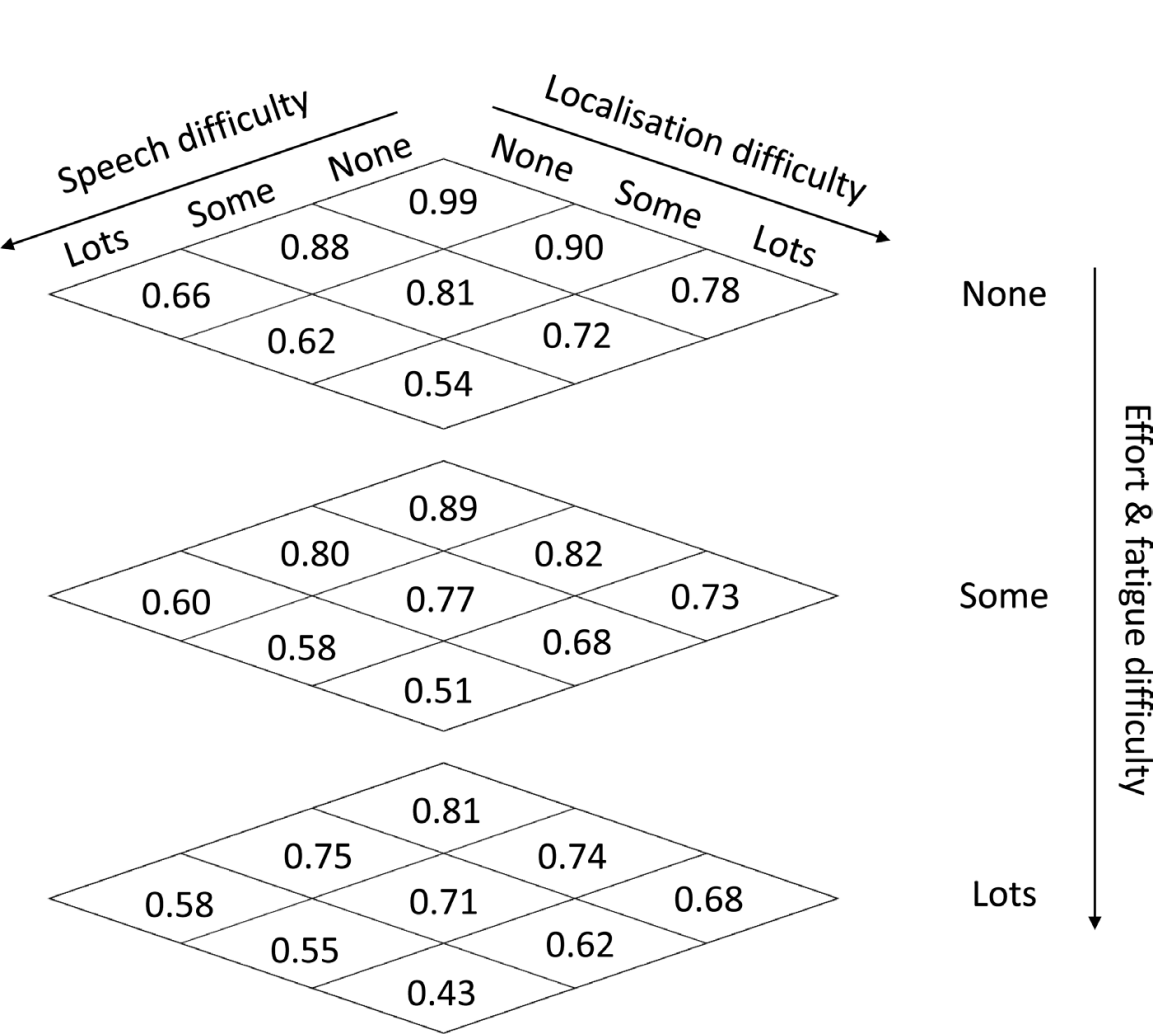
Mean utility values derived from the preferences of a sample of young adults for each of the 27 health states described in the YBHRQL-Y elicited using the time trade-off technique. YBHRQL-Y indicates York Binaural Hearing-Related Quality of Life questionnaire for Young People.

The mean health utility values assigned to the 27 hearing health states described by the YBHRQL-Y and directly considered by the 155 respondents are shown in Figure 2. The utility values range from 0.43 to 0.99 with the highest value assigned to the most advantageous health state and values decreasing monotonically as the level of difficulty is increased in any one domain or across a combination of more than one domain.

The pattern of utility values was consistent with the expectation that increasing the level of difficulty on any one domain would result in a decline in the perceived quality of that health state, and that describing difficulties across more than one domain would result in a larger decline in health than any of the individual difficulties separately. Linear regression mixed modeling confirmed that all three domains had a significant effect on the utility value [speech in noise, *F*(2, 4004) = 1049, *p* < 0.0001; localization, *F*(2, 4004) = 307, *p* < 0.0001; effort and fatigue, *F*(2, 4004) = 205, *p* < 0.0001].

### Reliability and Validity Assessments

The mean duration between administrations of the questionnaire sets was 17.1 days (SD = 4.7 days), with a minimum value of 14 days and a maximum value of 33 days. ICCs across time points (first administration versus second administration) were calculated for each domain and for the health utility values derived from the YBHRQL-Y. The health utility scores derived from YBHRQL-Y responses were found to have good test-retest reliability (ICC = 0.82, 95% CI = 0.71 to 0.89). Weighted Cohen’s Kappa values indicated that the categorical responses on each domain had at least moderate agreement (*κ* > 0.40) for the speech domain (*κ* = 0.52, 95% CI = 0.33 to 0.72), localization domain (*κ* = 0.52, 95% CI = 0.33 to 0.72), and the effort and fatigue domain (*κ* = 0.74, 95% CI = 0.59 to 0.89).

**Fig. 3.**
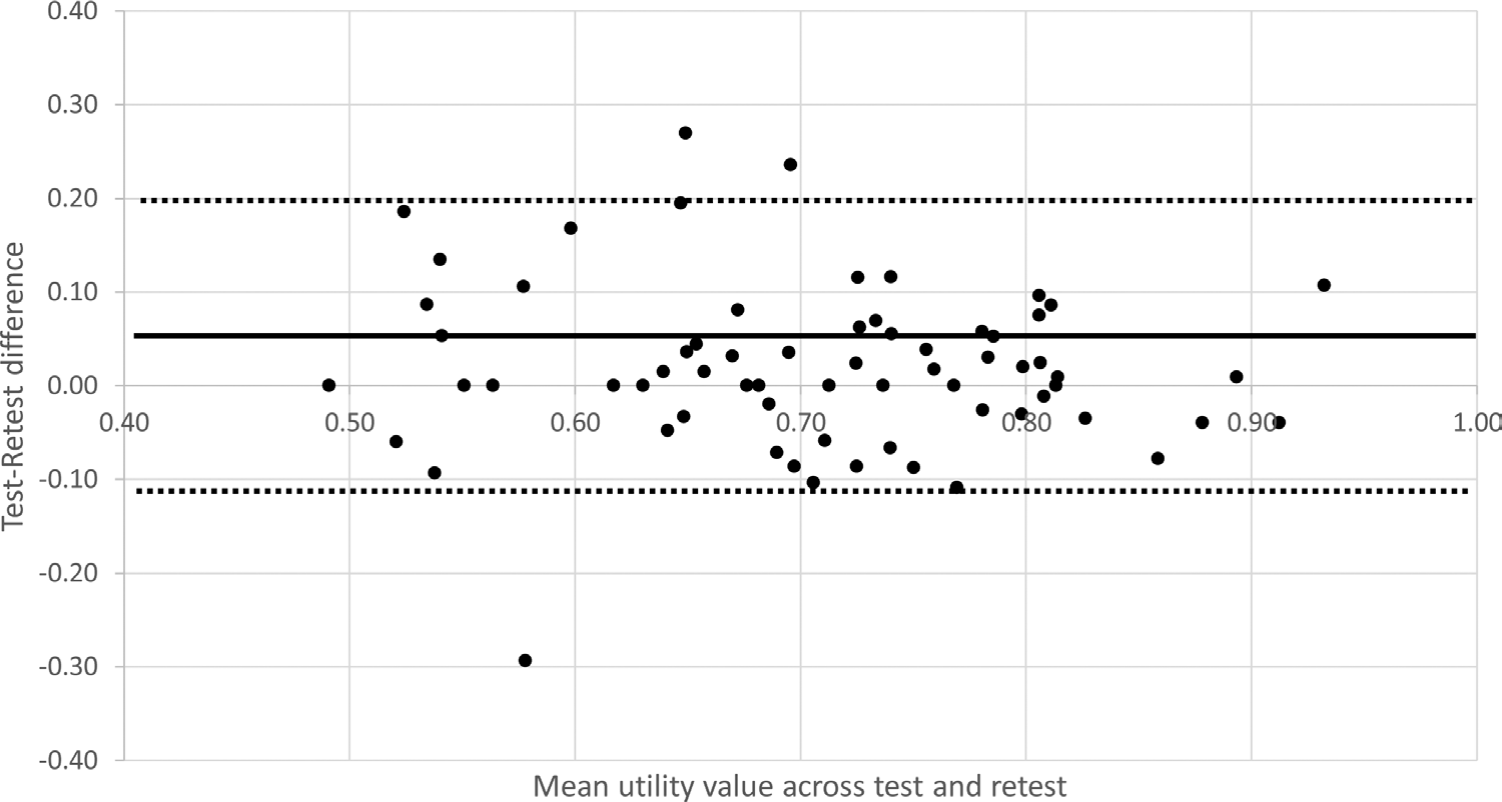
Bland-Altman plot of the mean test-retest utility scores from the child respondents (horizontal axis) plotted against the test-retest differences (vertical axis). Horizontal lines plot the mean test-retest difference (solid line) and 95% limits of agreement (dashed lines).

The Bland-Altman plot for the health utility values derived from the test-retest administrations of the YBHRQL-Y is shown in Figure 3. The plot reveals no systematic difference between runs with a mean test-retest difference of 0.018 (95% CI, −0.01 to 0.04) that did not differ significantly from zero (*p* > 0.05). The 95% limits of agreement covered the range between −0.15 and 0.19, and the variability of the test-retest differences appeared to be consistent across participants regardless of their mean health utility score.

Regression analyses suggested evidence for the construct validity of each individual domain. The categorical responses on the speech in noise domain were significantly associated with the speech in noise domain of the SSQ-Ch [*F*(4,66) = 7.293, *p* < 0.001, adjusted *R*^2^ = 0.26], as were the responses on the localization domain associated with the spatial domain of the SSQ-Ch [*F*(4, 66) = 3.894, *p* = 0.007, adjusted *R*^2^ = 0.14]. Finally, the categorical responses on the effort and fatigue domain were significantly associated with the VFS-Peds score [*F*(4, 66) = 18.13, *p* < 0.001, adjusted *R*^2^ = 0.49]. The YBHRQL-Y health utility values were also found to be significantly and positively associated with health utility values obtained using the HUI3 [*F*(1, 69) = 18.86, *p* < 0.001, adjusted *R*^2^ = 0.20] and the CHU9D [*F*(1, 69) = 13.12, *p* < 0.001, adjusted *R*^2^ = 0.15].

See Supplemental Digital Content (https://links.lww.com/EANDH/B708) for bubble plots illustrating the associations between: the speech in noise domain of the YBHRQL-Y and the speech domain from the SSQ-Ch; the localization domain of the YBHRQL-Y and the spatial domain of the SSQ-Ch; the effort and fatigue domain of the YBHRQL-Y and the VFS-Peds score; and finally, the health utility values derived from the YBHRQL-Y and the health utility values derived from both the HUI3 and CHU-9D.

### Feasibility of the Parent Proxy Version

The mean health utility values of the health states selected by the parents and guardians of the children (mean = 0.67, SD = 0.12) were lower than the states selected by the children themselves (mean = 0.72, SD = 0.11). This difference was found to be statistically significant [*t*(140) = 2.59, *p* < 0.05]. Further analyses indicated poor reliability between adult and child responses (ICC = 0.52, 95% CI = 0.22 to 0.70) and poor agreement between adult and child responses (*κ* values between 0.21 and 0.39).

## DISCUSSION

The YBHRQL-Y is an adapted version of a valid and reliable instrument for measuring binaural hearing-related quality of life in adults. The adapted version extends the application of this instrument to young people between the ages of 8 to 16 yr of age. The present analyses support both the construct validity of the individual domains of the YBHRQL-Y, which showed the hypothesized associations with established measures of each construct, and the overall utility weighted score derived from the preferences of young adults, which showed the hypothesized associations with generic measures of quality of life. Test-retest analyses suggested that both the raw responses and utility scores are reliable and show good agreement between repeated administrations. The sample recruited for the reliability and validity assessments included representation across the age range of the intended users of the YBHRQL-Y; that is, children from 8 to 16 yr of age. In fact, 21% of all participants were aged 8 yr old, the youngest age range the YBHRQL-Y is indicated for, providing further evidence that the psychometric properties of the instrument observed in the current study should be generalizable to even the youngest intended users.

A key feature of the YBHRQL-Y is its brevity. The YBHRQL-Y is able to define 125 hearing health states using only 3 short age-appropriate questions. This contrasts with other hearing-related measures in children such as the SSQ-Ch (33 items) and the VFS-Peds (10 items). The YBHRQL-Y is also shorter than gold standard measures of health-related quality of life used in pediatric research, the HUI3 (17 items) and the CHU 9D (9 items). The ability to administer an easily understood, valid, reproducible measure of hearing-related quality of life could be beneficial not only for researchers but also for pediatric audiologists and other professionals who see children with hearing loss in clinic.

An additional key feature of the YBHRQL-Y is its function as a tool within health economic analyses of hearing interventions. Health economic analyses are becoming more common in hearing research and clinical trials and are imperative to the widespread adoption of any new intervention or device. The brevity of the YBHRQL-Y means it can easily be incorporated into the development of hearing research in pediatrics to add the ability to conduct health economic analyses without adding substantial participant burden.

The current results provide evidence to suggest that the views of adult respondents about a child’s hearing-related quality of life may not agree with that of the child. Adults consistently ranked their children’s hearing-related quality of life to be worse than the child did, and there was both poor reliability and agreement across child and adult responses. This lack of agreement between adult and child is perhaps not surprising given the contexts set out in the YBHRQL-Y. For example, the instrument asks about a child’s experience in the “lunch hall,” when listening in an outdoor area at school break time, and about a child’s fatigue at the end of a school day. It could be anticipated that adults may have little-to-no experience of the child’s recent experiences in the first two contexts (the lunch hall and school break time), and so it is unsurprising that they are unable to accurately report a child’s lived experience in these contexts. Children and adults may also have differing views because adults may make comparative judgements based on their experiences of what other children can do with their hearing, whereas the child may only be able to place the level of difficulty in the context of their own personal experiences of listening. Nevertheless, the current results strongly suggest that if children are unable to complete the YBHRQL-Y, then it is not advisable to instruct an adult to complete the measure for them as the resulting measures may not be comparable.

## ACKNOWLEDGMENTS

The authors thank all the families that contributed to this work and made it possible to develop our measure. The authors thank Merle Mahon and Sandra Driver for their help with recruitment for this study.

## Supplementary Material
