# Supplementary material for: Validation and Preference-Based Scoring of the York Binaural Hearing-Related Quality of Life Questionnaire for Young People
Source: Ear Hear. 2025 Oct 30;47(1):202–9. doi: 10.1097/AUD.0000000000001713 (PMC12700680; doi:10.1097/AUD.0000000000001713)
Supplement: Supplementary file 1 [file aud-47-202-s001.pdf]

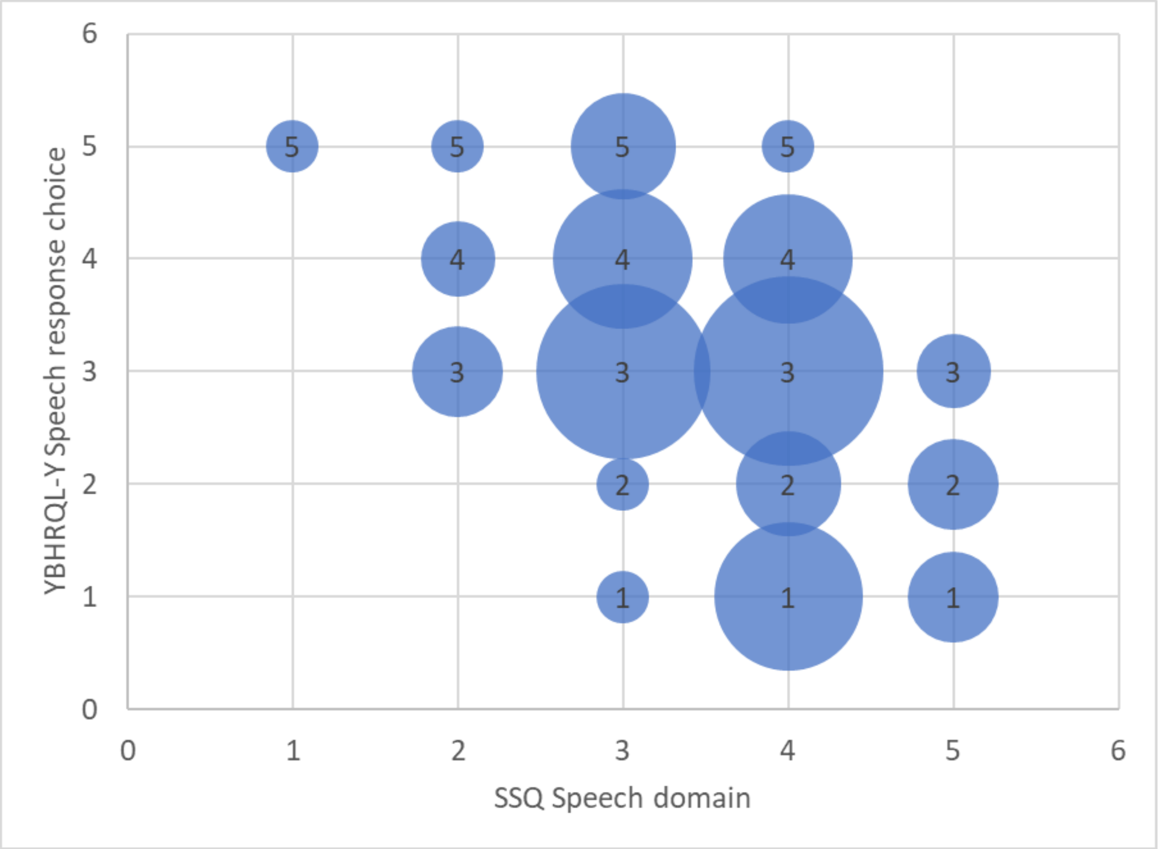

Bubble plot showing the association between the raw response choices on the speech domain question of the YBHRQL-Y (vertical axis) and the SSQ speech domain score when each child's score was categorized into one of 5 response categories dividing the numerical response range into 5 equal segments.

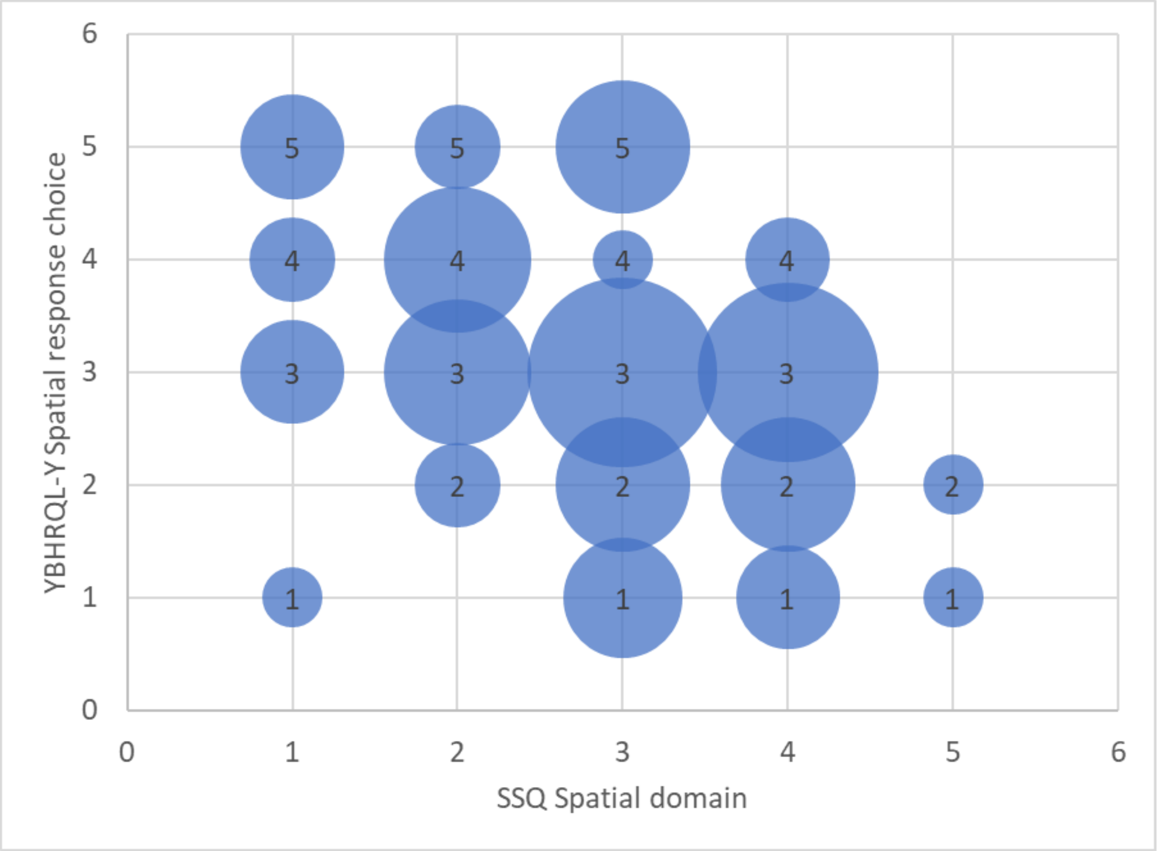

Bubble plot showing the association between the raw response choices on the spatial domain question of the YBHRQL-Y (vertical axis) and the SSQ spatial domain score when each child’s score was categorized into one of 5 response categories dividing the numerical response range into 5 equal segments.

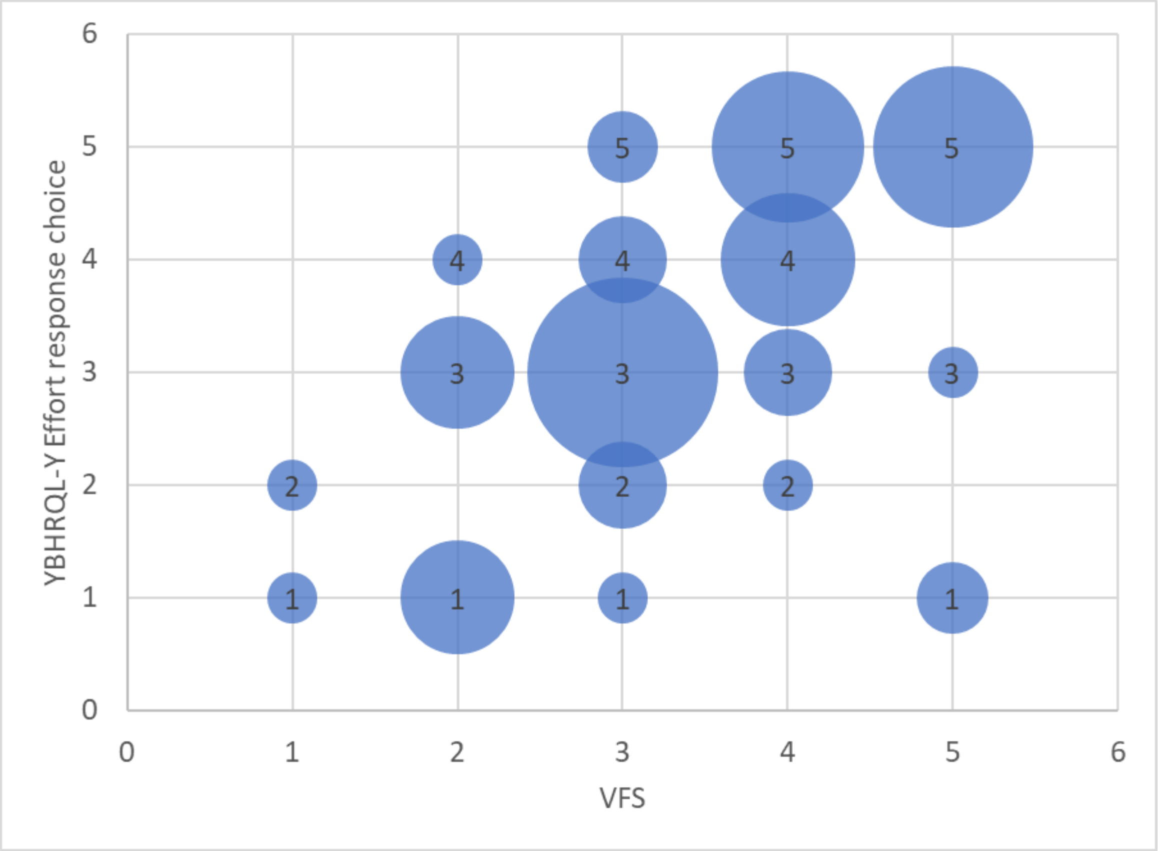

Bubble plot showing the association between the raw response choices on the effort domain question of the YBHRQL-Y (vertical axis) and the VFS score when each child's score was categorized into one of 5 response categories dividing the numerical response range into 5 equal segments.

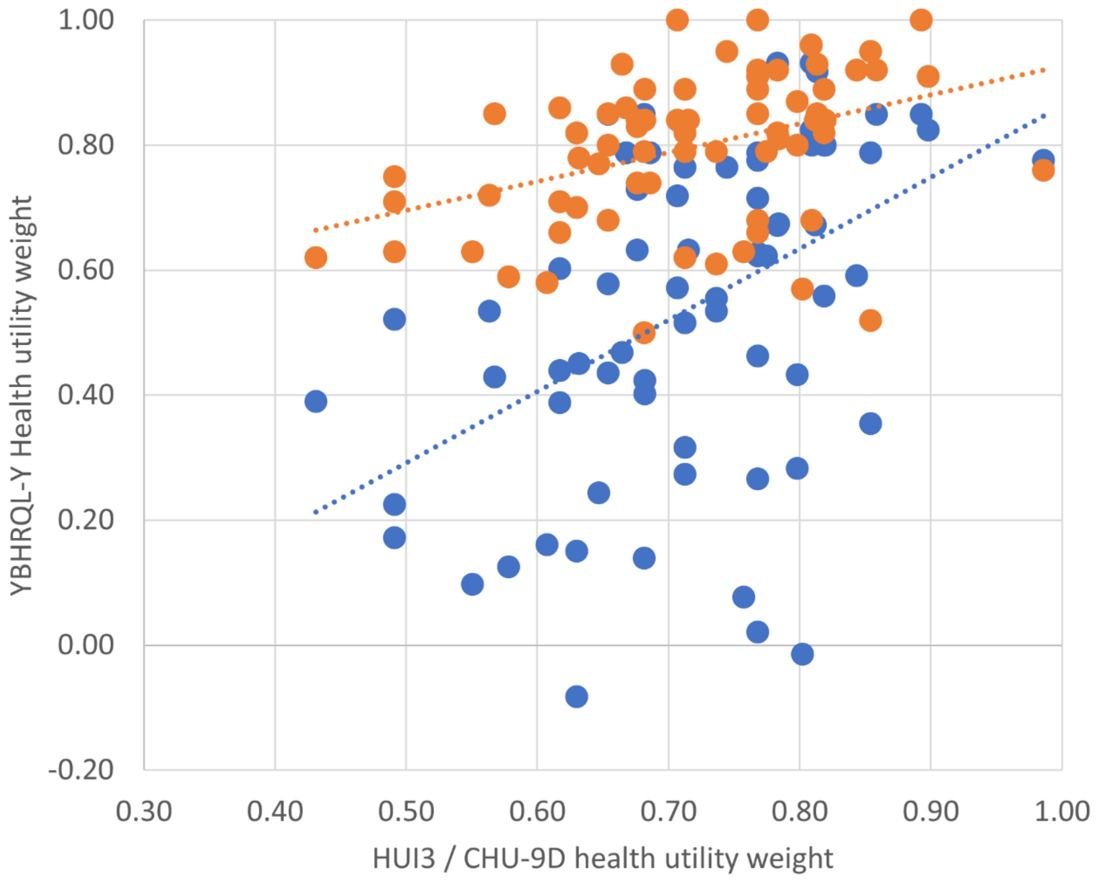

Scatter plot showing the relationships between the health utility weights derived using the YBHRQL-Y against both the HUI3 (blue) and the CHU-9D (orange). The dashed lines show the lines of best fit.
